# Polyphenol Stilbenes: Molecular Mechanisms of Defence against Oxidative Stress and Aging-Related Diseases

**DOI:** 10.1155/2015/340520

**Published:** 2015-06-09

**Authors:** Mika Reinisalo, Anna Kårlund, Ali Koskela, Kai Kaarniranta, Reijo O. Karjalainen

**Affiliations:** ^1^Department of Ophthalmology, University of Eastern Finland, P.O. Box 1627, 70211 Kuopio, Finland; ^2^Department of Biology, University of Eastern Finland, P.O. Box 1627, 70211 Kuopio, Finland; ^3^Department of Ophthalmology, Kuopio University Hospital, P.O. Box 1627, 70211 Kuopio, Finland

## Abstract

Numerous studies have highlighted the key roles of oxidative stress and inflammation in aging-related diseases such as obesity, type 2 diabetes, age-related macular degeneration (AMD), and Alzheimer's disease (AD). In aging cells, the natural antioxidant capacity decreases and the overall efficiency of reparative systems against cell damage becomes impaired. There is convincing data that stilbene compounds, a diverse group of natural defence phenolics, abundant in grapes, berries, and conifer bark waste, may confer a protective effect against aging-related diseases. This review highlights recent data helping to clarify the molecular mechanisms involved in the stilbene-mediated protection against oxidative stress. The impact of stilbenes on the nuclear factor-erythroid-2-related factor-2 (Nrf2) mediated cellular defence against oxidative stress as well as the potential roles of SQSTM1/p62 protein in Nrf2/Keap1 signaling and autophagy will be summarized. The therapeutic potential of stilbene compounds against the most common aging-related diseases is discussed.

## 1. Introduction

**Figure 1:**
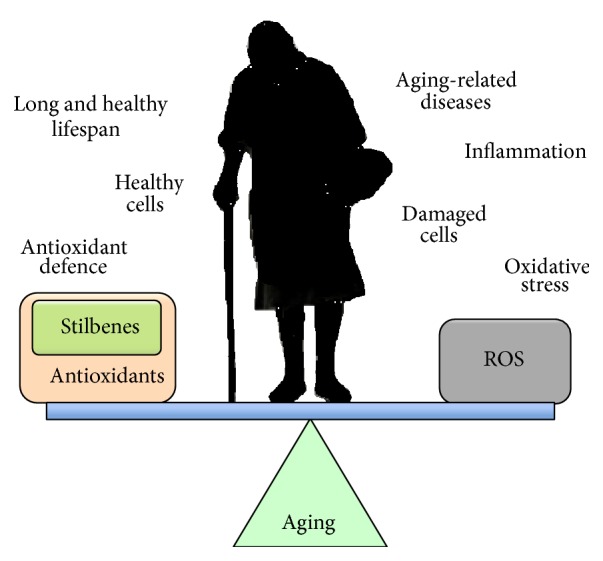
As an individual ages, the balance between a long, healthy lifespan and suffering age-related diseases is believed to be related to the interplay between the cellular antioxidant defence system and adverse effects related to oxidative stress. In aging cells, oxidative stress increases due to a progressive decline in the efficiency of antioxidant defence systems. There is convincing evidence to indicate that supplementation with polyphenols, such as stilbenes, anthocyanins, and catechins, can increase cellular antioxidant defence and promote health of the individual.

Aging is one of the major risk factors for a wide range of chronic, metabolic, and neurodegenerative diseases. During the course of aging, reactive oxygen species (ROS) cause increasing oxidative stress in cells leading to oxidative damage and to the induction of inflammatory cascades (Figure 1). Furthermore, antioxidant defence systems against oxidative stress deteriorate during aging and many antioxidant phase II genes are upregulated only marginally or may even be downregulated. Moreover, the innate and adaptive immune defence systems tend to deteriorate during aging and this may contribute to the degeneration process [1].

Activation of antioxidant defence and phase II enzymes is a key endogenous mechanism protecting cells from the oxidative damage associated with many common diseases such as obesity, type 2 diabetes (T2D), age-related macular degeneration (AMD), and Alzheimer's disease (AD). The activation of cellular defence mechanisms by plant-derived bioactive compounds is believed to attenuate cellular oxidative stress and thus this seems to represent a feasible therapeutic approach against age-related diseases. There is increasing epidemiological and experimental data suggesting that the regular intake of berries, vegetables, and fruits containing significant amounts of polyphenols is a potential way to improve the quality of life as an individual grows older [2–4]. Polyphenols or polyphenol metabolites produced in the body mediate their protective action via several mechanisms and different pathways that are currently under intensive study. For example, the beneficial effects of anthocyanin-rich bilberry and blackcurrant diets, proanthocyanidin (later referred to as PAC) metabolites, and grape-derived polyphenolic preparations have been extensively studied not only in animal models but also in patients with T2D and AD [5–7].

Stilbene compounds are part of a vast group of natural defence polyphenols occurring in many plant species. Resveratrol (3,5,4′-trihydroxy-trans-stilbene) is a well-known polyphenol phytoalexin which is found mainly in the skin of grapes; it has attracted extensive scientific attention due to its potential health benefits related with its cardiovascular (French paradox), chemopreventive, antiobesity, antidiabetic, and neuroprotective properties. However, recent data have highlighted that also other stilbene compounds such as pterostilbene (3,5-dimethyl ether derivative of resveratrol) may have higher bioavailability and possess better neuroprotective activity against AD than resveratrol itself [8]. Another interesting stilbene compound with potential health-beneficial properties is pinosylvin (3,5-dihydroxy-trans-stilbene), a naturally occurring trans-stilbenoid which is mainly found in the heartwood of* Pinus* species and occurs in high concentrations in bark waste and thus this stilbene compound may represent an inexpensive polyphenol with considerable potential for diverse health-promoting applications [9–11].

There are several comprehensive reviews available focusing on resveratrol, but very few reports have analyzed the bioactivity of other stilbenes. Here, we review shortly the most recent data to clarify the molecular mechanisms involved in stilbene-mediated protection against oxidative stress, emphasizing the potential roles of transcription factor nuclear factor-erythroid-2-related factor-2 (Nrf2) and SQSTM1/p62 protein (later referred to as p62) in the regulation of antioxidant enzymes and autophagy. The therapeutic potential of stilbene compounds to target the molecular pathways behind many common aging-related diseases will be reviewed.

## 2. Stilbene Compounds in Plants

Phenolic compounds are important mediators of adaptation and survival responses of plants in acute and chronic challenges, but polyphenols also act in plants regulating cell growth, differentiation, pollen fertility, and nodulation and thus seem to be essential for plant health. For example, stilbenes are natural phenolic defence compounds occurring in a number of different plant species that possess antimicrobial and antioxidant activities against phytopathogens and ozone or UV stress [12].

Stilbene compounds occur in many plant species (Table 1) including grape wine (*Vitis vinifera*), peanut (*Arachis hypogaea*), sorghum (*Sorghum bicolor*) and many tree species (*Pinus* and* Picea*) [13]. Moreover, commercial sources of stilbenes include many plants cultivated in Asia as folk medicines such as* Polygonum cuspidatum, Rhodomyrtus tomentosa, Rheum undulatum, Melaleuca leucadendron*, and* Euphorbia lagascae*, while pterostilbene is found predominantly in bilberries (*Vaccinium myrtillus*), blueberries (several* Vaccinium* species), and some other berries as well as in grapes and juice residues which are important source of this stilbene when it is used in nutraceutical applications [14]. Grape pomaces, residues produced during wine making, and other grape juice solids contain high polyphenol concentrations and are also attractive sources of many stilbene compounds not only resveratrol [15]. The bark waste of conifer trees contains substantial amount of stilbene compounds such as pinosylvin, piceatannol, and* trans*-resveratrol (*t*-Res). Thus, this enormous amount of industrial byproducts represents a very attractive and inexpensive source of stilbenes with commercial applications [16, 17]. Genetic tools are a very promising means to produce specific stilbenes such as pterostilbene via stilbene synthase and* O*-methyltransferase coexpression in plants [18]. These types of stilbenes may be especially suitable for pharmacological applications. The major stilbenes and their structures are described in Table 1 and a more complete list can be found in reviews [12, 19].

Stilbene synthase (STS) is the key enzyme that catalyzes the biosynthesis of stilbenic compounds. STS has evolved from the chalcone synthases (CHSs) apparently several times independently in stilbene-producing plants [13, 19]. Interestingly, different STS genes display different tissue and developmental specific expression. Thus, it has been reported that STS genes exhibited lower expression levels in young grape leaves than in old leaves while the transcript levels of eight STS genes increased dramatically in the berry skin of grape cultivars Cabernet Sauvignon and Norton post véraison reaching the highest level at the time of harvest [2]. The heartwood of pine trees contains high level of pinosylvin, but as a response to stress induction (fungal or UV light), young seedlings also accumulate high amounts of pinosylvin [20].

## 3. Bioavailability

At least a proportion of the stilbene compounds or their metabolites present in extracts may be sufficiently bioavailable to reach even brain target cells and thus exert beneficial actions [7, 34]. Although the oral absorption of resveratrol in humans has been claimed to be as high as 75% [35], it is well known that stilbenes, however, are poorly bioavailable phenolic compounds after oral intake. Due to extensive metabolism in the major sites in intestine and liver (glucuronides and sulfates are the major metabolites of resveratrol), the oral bioavailability appears to be less than 1% in a rat model. The oral bioavailability of rhaponticin was calculated to be 0.03% [36]. In another rat study, following oral dosing, plasma levels of pterostilbene and pterostilbene sulfate were markedly greater than the levels of resveratrol and resveratrol sulfate indicating that the* in vivo* biological activity of equimolar doses of pterostilbene may be greater than that of resveratrol [37]. Moreover, the absolute oral bioavailability of pterostilbene was found to be around 12% in rat plasma with the values of terminal elimination half-life and clearance of pterostilbene being 96.6 ± 23.7 min and 37.0 ± 2.5 mL/min/kg suggesting that bioabsorption is very rapid with peak concentration achieved at 0.5–2 h after the oral dose and excretion being complete a few hours after ingestion [38].

In a human trial, 10 healthy volunteers received single doses of 0.5, 1, 2.5, or 5 g resveratrol; the peak levels of resveratrol and six metabolites at the highest dose were 539 ± 384 ng/mL (2.4 micromol/L, mean ± SD; *n* = 10) analyzed 1.5 h after dose [39]. Interestingly, the area under the plasma levels curve (AUC) values for resveratrol-3-sulfate and resveratrol monoglucuronides was up to 23 times greater than that of resveratrol. In the other human trial performed in 40 healthy volunteers, repeated doses of resveratrol were tested with the volunteers ingesting 29 daily resveratrol doses of 0.5, 1.0, 2.5, or 5.0 g [40]. The data revealed that resveratrol-3-*O*-sulfate, resveratrol-4′-*O*-glucuronide, and resveratrol-3-*O*-glucuronide were the major plasma metabolites. Maximal plasma levels and areas under the concentration versus time curve for the metabolites exceeded the levels of resveratrol by about 20-fold. When resveratrol at doses of 0.5 or 1.0 g was given to 20 patients suffering from colorectal cancer, both resveratrol and resveratrol-3-*O*-glucuronide were recovered from tissues at maximal mean concentrations of 674 and 86.0 nmol/g, respectively [41]. Interestingly, it was claimed by the authors that these daily doses of 0.5 or 1.0 g produced levels in the human gastrointestinal tract at order of magnitude sufficient to elicit anticarcinogenic effects.

Low bioavailability, poor solubility, limited stability, high rate of metabolic breakdown, and low target specificity have been considered as major obstacles to the use of resveratrol and its natural analogies in major pharmacological applications. However, several research lines are currently underway to improve these properties [42]. A wide range of synthetic derivatives of resveratrol have been generated, and some of the best derivatives have improved the target specificity down to the nanomolar range [43]. There is a remarkable finding that inhibition of aromatase activity was enhanced by over 6000-fold when the central ring of resveratrol was substituted with 1,3-thiazole; this suggests that this modified resveratrol may be a potential drug for treating breast cancer.

Recently it was reported that soluble galenic form improved low absorption of* t*-Res as a dry powder [44]. The efficacy of the new formulation was tested in 15 healthy volunteers receiving 40 mg of* t*-Res. The single dose (40 mg) of the soluble* t*-Res was found to be well absorbed and elicited biologically efficient blood levels (0.1–6 *μ*M) for several hours; the new soluble galenic-based formulation led to 8.8-fold higher resveratrol levels in plasma versus the control powder. Interestingly, this new formulation elicited an intense anti-inflammatory response in various tissues of mice fed a high-fat diet (HFD) while the control diet exhibited only a weak response suggesting that improved plasma bioavailability confers significant enhancement of biological activity in the target cells. In another recent work, a modification of the liposome with polyethylene glycol (PEG) was used to improve the bioavailability of rhaponticin (RA) and its plasma protein binding ability [45]. This experiment revealed that the maximum plasma concentration (*T*_max⁡_) of PEGL-RA was about 4.5 times higher than that of free RA after oral administration due to the lower distribution into the gastrointestinal tract. Addition of piperine alkaloid (100 mg/kg; oral gavage) + piperine (10 mg/kg; oral gavage) in a mouse trial lead to a substantial increase (1544%) in the maximum serum concentration (*C*_max⁡_) as compared with the standard resveratrol dose [46]. Recently, it was reported that a higher concentration of resveratrol could be achieved in the brain tissue by administrating the compound inside lipid-core nanocapsules [47].

The safety of food ingredients (dietary supplements, functional foods, etc.) containing substantial amounts of polyphenols is an important issue. In the majority of studies, stilbene compounds such as resveratrol have appeared to be well tolerated and no marked toxicity has been reported [48]. In a human trial, 25, 50, 100, or 150 mg of* t*-Res was given six times a day, but adverse events were mild in severity and similar between groups [49]. Only at very high doses used in some human studies such as repeated doses at 2.5 and 5 g levels have there been reports of mild to moderate gastrointestinal symptoms [48], but even such high levels given as single doses did not cause any adverse events [39, 40]. However, stilbene-drug interactions have not been clarified and remain to be determined.

Gastrointestinal symptoms at a 1 g daily dose were also observed and it has been suggested that 1 g of daily resveratrol dose should not be exceeded in clinical trials [40]. Moreover, administration of 2 g* t*-Res twice a day was recently found to achieve adequate biological exposure and it was well tolerated in healthy subjects, although diarrhea was frequently observed; thus, it was proposed that to maximize* t*-Res exposure and safety these supplements should be taken with a standard breakfast and not with a high-fat meal [50].

## 4. Molecular Basis of Oxidative Stress

### 4.1. The Nrf2/ARE Pathway in Cellular Defence

**Figure 2:**
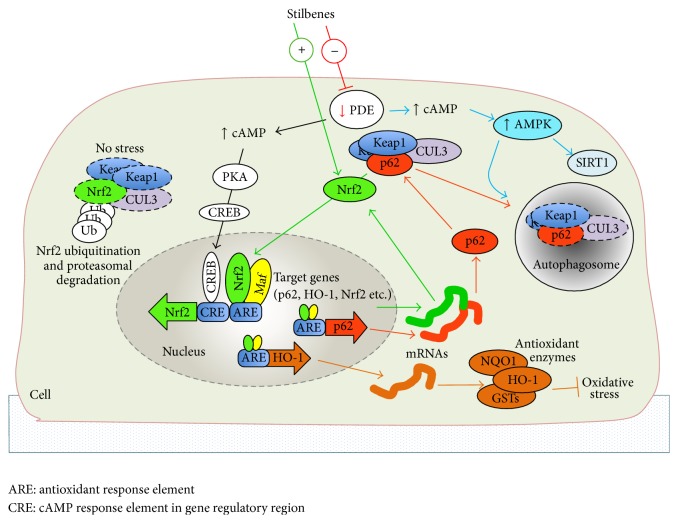
The Nrf2/ARE pathway and cAMP second messenger system together are the key regulators of cellular antioxidant defence. These pathways can be modulated by stilbenes. Stilbenes can activate nuclear localization of Nrf2 and activation of Nrf2 target genes associated with antioxidant defence and autophagy. Autophagy related protein p62 and Nrf2 form a regulatory loop where p62 enables the release of Nrf2 from cytoplasmic Keap1 complex. When cells are not stressed, the excess of cytoplasmic Nrf2 is eliminated by proteasomal degradation. In addition, stilbenes are capable of activating cAMP response element-binding protein (CREB) target genes and the AMPK pathway by PDE inhibition mediated increase of cellular cAMP levels.

Nuclear factor-erythroid-2-related factor-2 (Nrf2), a member of the basic leucine zipper (bZIP) transcription factor family, is an essential transcription factor for cellular detoxification and defence against oxidative stress. In the cell nucleus, Nrf2 is able to recognize the antioxidant response element (ARE) with the specific nucleotide binding sequence (5′-TGACnnnGC-3′) positioned in regulatory region of target genes [51, 52]. Role of other members of the Nrf-family such as Nrf1 and Nrf3 has not been so thoroughly studied but current evidence suggests that these genes have partially different functions, target genes, and tissue-specificities although they recognize the same ARE sequence as Nrf2 [51, 53]. By acting through the ARE element, Nrf2 has a central role in the regulation of a large group of phase II metabolite conjugation and antioxidant genes (Figure 2) as well as in influencing some of the genes involved in proteasome pathway and inflammation [52, 54–57]. Until now, several Nrf2 target genes (see Table 2) such as heme oxygenase-1 (HO-1) [58] and NAD(P)H dehydrogenase, quinone 1 (NQO1) [59], have been verified. Under basal conditions, the Nrf2/ARE pathway is suppressed since Nrf2 is trapped in the cytosol as it forms a protein complex with Kelch-like ECH-associated protein 1 (Keap1) [55]. Keap1 acts as a molecular switch sensing cellular electrophile and oxidant homeostasis [60]. With assistance of Cullin-3 (CUL3), the Nrf2-Keap1-CUL3 protein complexes are constantly exposed to ubiquitin conjugation and proteasomal degradation [55, 61]. In condition of stress or exposure to electrophiles, Nrf2 dissociates from the Keap1-CUL3 complex and translocates into the nucleus. The dissociation of Nrf2 is mediated via modification of specific Keap1 cysteine residues by electrophiles, oxidants, and dietary supplements such as stilbenes [62]. Alternatively, dissociation of Nrf2 from cytoplasmic Nrf2-Keap1-CUL3 complex is enabled by p62 involved in autophagy process (see Section 4.2). In the nucleus, Nrf2 heterodimerizes with small Maf (sMaf) proteins which seems to be indispensable partners required for ARE binding and subsequent transactivation of target genes [52]. In contrast, in the nucleus, the transcriptional repressor BACH1 seems to have an important role as an antagonist for Nrf2 mediated activation by binding ARE-like elements in Nrf2 target genes [63]. It should be noted that Keap1 has the capability to undergo nuclear localization and to shuttle back to cytoplasm; this suggests that Keap1 is also involved in the regulation of Nrf2 in the cell nucleus [64]. Interestingly, in different species, ARE elements are also found in the regulatory regions of Nrf2 itself as well as in several regulators of the Nrf2/ARE pathway such as Keap1, sMaf, and p62 [65, 66]. In addition, it has been shown that the acetylation-deacetylation status of Nrf2 in the nucleus is also important for Nrf2 binding and target gene activation [67]. Auxiliary mechanisms such as the cAMP/CREB pathway [68] and the aryl hydrocarbon receptor (AhR) pathway [69] interacting with the Nrf2/ARE pathway will be discussed later in this review (see Section 4.3).

Convincing evidence from knockout animal models has proved that Nrf2 and Keap1 regulate numerous cellular functions [70, 71]. For example, Nrf2 knockout mouse displays progressive degeneration of retina (see Section 5.3) characteristic for AMD [71]. In Keap1 knockout mouse excessive accumulation of Nrf2 into nucleus stimulates aberrant expression of Nrf2 target genes causing growth retardation and the death of pups soon after birth [54]. The dramatic changes in growth indicate that Keap1 has an essential role in Nrf2 regulation and expression of Nrf2 target genes. For instance, Keap1 knockout mice displayed a constant overexpression of cytoprotective proteins such as phase II enzymes NQO1 and glutathione S-transferases (GSTs). Moreover, recent genome-wide studies have revealed numerous putative Nrf2 target genes [52, 72] suggesting that the Nrf2/ARE pathway may possess several novel molecular targets for polyphenols still to be found.

### 4.2. Role of p62 Protein in Nrf2/ARE Signaling and Autophagy

Current data indicates that Nrf2 is involved in autophagy, a catabolic process activated during starvation. For instance, in the retinal pigment epithelial (RPE) cells of the Nrf2 knockout mouse eye, the autophagy process and lysosome-dependent degradation were disturbed with accumulated autophagy intermediates, photoreceptor outer segments (POS), and an aging pigment, lipofuscin [70]. Autophagy supplies an energy resource of amino acids and other substrates via lysosomal degradation and recycling of unnecessary cellular components [88]. The impaired autophagy system has been shown to associate with aging-related neurodegenerative diseases such as Parkinson's disease (PD) [89], AMD [90], and AD [91]. One sign of this impairment is the accumulation of autophagy receptor p62 in AMD [90]. In addition, p62 is a multifunctional protein involved in other cellular functions such as bone metabolism, inflammation, and adipogenesis [92–94]. In order to eliminate cellular waste and protein aggregates during nutrient deprivation, the cell may trigger the complex autophagy process where the p62 protein has a central role [88]. In an experiment conducted in autophagy deficient mice it was found that p62 is involved in the formation of cellular protein aggregates which are normally eliminated by autophagy [95]. There is growing evidence that p62 is also capable of interacting with the Nrf2/ARE pathway (Figure 2) by disrupting the cytoplasmic Nrf2-Keap1 complex [66]. Moreover, it has been shown that the functional ARE element is located in regulatory region of p62 gene [52, 66]. Nrf2-p62 couple seems to form a regulatory loop where Nrf2 is able to activate p62 expression and consequently Nrf2 nuclear localization is facilitated by p62 [96, 97]. Interestingly, there is data that p62 is able to bind on specific Keap1 motif required for Nrf2 binding [66, 96] and that Keap1 elimination is processed by p62 dependent autophagy [97]. This was also shown in autophagy deficient mouse where Nrf2 became accumulated in cell nucleus [95]. However, the nuclear localization of Nrf2 was diminished when p62 was abolished.

It has been shown that AMP-activated protein kinase (AMPK) is the key regulator of autophagy [97] suggesting that the AMPK pathway is also likely involved in the regulation of p62 and the Nrf2/ARE pathway (Figure 2). AMPK is able to induce autophagosome formation by activating numerous downstream kinases and interacting proteins such as autophagy-related proteins, protein kinase ULK1, and microtubule-associated protein LC3 [93, 98] finally achieving the oligomerization of p62 within autophagosomes [99]. The activation of autophagy is concurrently aided by AMPK mediated inhibition of mammalian target of rapamycin (mTOR), a known suppressor of autophagy [100]. In contrast, mTOR is capable of inhibiting autophagy via phosphorylation of ULK1 [101]. It is known that both AMPK and autophagy are activated upon starvation. This pathway has been verified by utilizing 5-aminoimidazole-4-carboxamide-1-b-D-ribofuranoside ribonucleoside (AICAR), an activator of AMPK, autophagy, and NAD-dependent deacetylase sirtuin 1 (SIRT1) [90, 102–104]. For instance, AICAR mediated activation of the AMPK pathway can increase HO-1 expression via Nrf2 [104]. Interestingly, also stilbenes such as resveratrol can facilitate AMPK activation and autophagy [105].

### 4.3. Modulation of Other Pathways Underlying the Antioxidant Defence Systems

There is growing data that polyphenols have alternative molecular targets and this complicates elucidation of their role in cellular physiology and pathophysiology. Recently, interesting information regarding novel molecular targets of stilbenes such as cellular cAMP second messenger signaling, the AMPK pathway regulating energy homeostasis, estrogen-related receptor alpha (ERR*α*), and estrogen receptors (ER) as well as the enzymatic cofactor tetrahydrobiopterin (BH4) has become a focus of attention. Apparently, some of these targets may interact with the Nrf2/ARE pathway and the autophagic process.

*Stilbenes Can Restore the Cellular Bioavailability of the Enzymatic Cofactor Tetrahydrobiopterin (BH4) under ROS Exposure.* There is evidence that ROS are able to decrease the bioavailability of BH4 and coupling to aromatic amino acid hydroxylases, enzymes essential in many of the metabolic pathways involved in vascular and neurotransmitter homeostasis [106]. It is noteworthy that BH4 is an essential cofactor of nitric oxide synthase enzymes (NOS) involved in nitrogen oxide (NO) synthesis in almost all tissues [107, 108]. It has been shown that in NO synthesis, the lack of BH4 initiates superoxide generation and further synthesis of the powerful oxidant, peroxynitrite [109]. Moreover, in the brain and the eye, BH4 is required for the synthesis of tyrosine, dihydroxyphenylalanine (L-DOPA), dopamine, and serotonin [110] by acting as an essential cofactor of the key enzymes phenylalanine hydroxylase, tyrosine hydroxylase, tyrosinase [111], and tryptophan hydroxylase [112], respectively. Interestingly, BH4 is also pivotal for synthesis of two important neurotransmitters, norepinephrine and epinephrine [113], and serotonin [114]. It is noteworthy that serotonin derived melatonin can also act as an efficient free radical scavenger in the eye [115]. Therefore, it can be concluded that by increasing cellular BH4 levels, stilbenes can also contribute to the production of endogenous antioxidants. It has been shown in cell and animal models that resveratrol can increase BH4 synthesis and decrease the oxidation of BH4 [116]. In condition of oxidative stress, cellular BH4 stores are diminished and this disturbs several physiological functions [110]. It has been proposed that BH4 deficiency due to oxidative stress is associated with PD and AD [110] whereas the role of BH4 in AMD is unclear. However, in the retina, BH4 may have an essential role in regulation of retinal neovascularization via L-DOPA, since L-DOPA is capable of controlling levels of vascular endothelial growth factor (VEGF) via secretion of antiangiogenic pigment epithelial derived factor (PEDF) [117, 118].

*Stilbene Mediated Activation of the cAMP Pathway Contributes to Autophagy and Defence against Oxidative Stress.* It is known that resveratrol can increase cAMP levels in cells and animal models by their ability to inhibit cAMP phosphodiesterase [105]. Phosphodiesterases (PDE) are the enzymes responsible for degradation of cAMP and cGMP [119], second messengers involved in the regulation of numerous genes and cellular functions.

Recent findings have verified that activation of the cAMP signaling targets by stilbenes such as resveratrol can influence important functions in aging cells associated with antioxidant defence. First, cAMP is able to induce Nrf2 expression in cells [120]. Similarly, activation of Nrf2 via cAMP can be induced by *α*-melanocyte stimulating hormone (*α*-MSH), a known hormonal activator of G protein-coupled melanocortin receptors and the cAMP pathway [68]. Apparently, activation of Nrf2 transcription by cAMP is mediated via protein kinase A (PKA) and cAMP response element-binding protein (CREB). In the presence of cAMP stimuli, CREB can transactivate Nrf2 as well as Nrf2 target genes such as glutathione S-transferase pi (GSTP1) and NOS [121, 122] by binding on specific cAMP response element (CRE) in promoter region [123]. Second, by inhibiting PDE activity resveratrol is able to increase cellular cAMP and Ca^2+^ levels, finally activating the AMPK pathway involved in the regulation of nutrient homeostasis [105, 124] such as autophagy as described in Section 4.2. The AMPK can increase cellular levels of NAD^+^ as well as the activity of SIRT1 [105, 125]. It has been shown that SIRT1 can modulate the activity of metabolic regulators peroxisome proliferator-activated receptor *γ* coactivator-1*α* (PGC-1*α*) and ERR*α* [102, 126]. However, information regarding the interaction between the AMPK and Nrf2/ARE pathways is contradictory. It was shown recently that known AMPK pathway inducers AICAR and berberine can also induce the Nrf2/ARE pathway [104, 127], whereas in contrast it was shown that deacetylation of Nrf2 by SIRT1 decreases DNA binding activity of Nrf2 causing decreased promoter activity of Nrf2 targets such as HO-1 and NQO1 [67]. This was also verified with resveratrol which was shown to act as a SIRT1 activator.

*Polyphenols Are Involved in Regulation of Nuclear Receptors (NRs).* The large family of NRs including nuclear hormone receptors and orphan receptors with somewhat unknown ligands is associated with multiple functions in the human body, from development to hormonal regulation and metabolism. In particular, NRs are also involved in the metabolism of xenobiotics [128, 129] and therefore NRs and their targets are under intense investigation in drug industry. Interestingly, current data suggests that several polyphenolic compounds can activate different NRs. This seems reasonable since the Nrf2/ARE pathway and some NRs are activated by phytochemicals and xenobiotics and they are thought to participate with regulation of xenobiotic metabolizing enzymes such as CYP3A4 and NQO1 [69, 130, 131]. In such interactions, polyphenols can act as a ligand for nuclear receptor (resveratrol/ERs) or activation of NRs is mediated via Nrf2 as has been found with retinoid X receptor alpha (RXRa) [72]. For instance, Nrf2 has been shown to bind aryl hydrocarbon receptor (AhR) promoter, and vice versa, AhR element is found from Nrf2 promoter [69]. AhR is involved in cytokine and growth factor signaling and particularly it is a regulator of several xenobiotic metabolizing enzymes activated by exogenous ligands such as dioxins [132]. Furthermore, Nrf2 and AhR have shared target genes such as NQO1, GSTs, and UDP-glucuronosyltransferases (UTGs) [78, 133]. It should be noted that activation of UTG1A8 and UTG1A10, phase-II enzymes involved in glucuronidation mediated elimination of xenobiotics, requires cooperative induction via both factors AhR and Nrf2 [81]. Moreover, activation of NRs can also be mediated via kinase pathways such as the AMPK-SIRT1 pathway in case of ERR*α* regulation [102].

*Estrogenic Activity of Stilbenes.* Interestingly, it seems that some beneficial effects of stilbene derivatives in neuronal and vascular cells are mediated via ERs, typically activated by estrogens (17*β*-estradiol) [134]. In addition to estrogens, ERs can also bind nonsteroidal compounds such as phytoestrogens [135]. For instance, the ER-specific agonist functions of resveratrol are thought to be attributable to its structural similarities with estrogens; thus resveratrol and some stilbene derivatives can be classified as selective ER modulators (SERMs) [135, 136]. Recently, it has been shown that resveratrol can act through estrogen receptors (ER*α* and ER*β*) to exert neuroprotective activity [137] and it can increase the expression of the dopamine transporter (DAT) in dopaminergic cells [138]. Moreover, it seems that ERs can also mediate the beneficial effects of resveratrol in vascular cells such as vasodilatation by increasing cGMP synthesis, eNOS activity, and NO production [106].

## 5. Therapeutic Potential of Stilbenes against Oxidative Stress and Age-Related Diseases

### 5.1. Obesity

Obesity is a major global health problem; for example, it is one of the major risk factors for T2D. The western life style including high energy food intake and inadequate physical activity is the cause of adipocyte dysfunction leading to the storage of extra energy as triglycerides. The release of proinflammatory cytokines from visceral adipose tissue, liver insulin resistance, and inflammation lead to an increasing risk of several metabolic diseases [139]. Prevention of extra energy storage via caloric restriction is a well-documented means to reduce obesity, to increase the life span [140–142], and even to prevent the memory decline [143]. However, the current obesity problem indicates clearly that, although lifestyle changes are effective in practice, they are very difficult to achieve. The molecular mechanism of obesity has not been fully clarified, but an increase in the number and size of adipocytes differentiated from preadipocytes in mature adipocytes seems to be a key pathway in the route towards [144]. It has long been known that resveratrol can mimic some of the impacts of calorie restriction (CR), but stilbene compounds may mediate their antiobesity action also by reducing the synthesis of lipids in adipocytes, modulating of lipolysis, and reducing inflammation and oxidative stress in the target tissue [140]. Although there are abundant data suggesting that stilbene compounds such as resveratrol may increase lifespan through the modulation of insulin signaling even on a high-calorie diet, the practical outcomes of these findings is far from clear [145, 146]. Moreover, comparison of the effectiveness of CR and resveratrol to the HFD-induced obesity and fatty liver formation in C57Bl/6J mice lead to the finding that CR provided superior protection against diet-induced obesity and fatty liver formation compared with resveratrol [147].

Stilbene compounds presumably act on several molecular targets in adipocytes eventually leading to the decreasing levels in adipocyte number and size. With respect to the recently synthesized several stilbene analogues, 3-hydroxy-trans stilbene inhibited adipocyte differentiation and enhanced glucose uptake resulting in a reduction of obesity [148]. The impact of resveratrol on fat cell apoptosis has not been intensively studied, but it has been reported that resveratrol inhibited human preadipocyte proliferation and adipogenic differentiation in a SIRT1-dependent manner and* de novo* lipogenesis was inhibited in parallel with a downregulation of lipogenic gene expression [149]. SIRT1 may widely regulate fatty acid oxidation in liver, fat mobilization in white adipose tissue, insulin secretion in pancreas, and sense nutrient availability in hypothalamus [150]. However, resveratrol reduced fat cell number also via SIRT1-independent mechanism [151]. Thus, the apoptotic effects of stilbenes in 3T3-L1 preadipocytes may be complex and involve several pathways such as AMPK, AKT, and survivin [152].

Peroxisome proliferator-activated receptor-*γ* (PPAR*γ*) is an important regulator of lipid and energy metabolism as well as one of key factors in the differentiation of adipocytes. In a microarray analysis it was demonstrated that there were changes identified in 35 genes involved in the PPAR*γ* signaling pathway, lipid metabolism, or adipogenesis in adipocytes treated with grape seed extract (GSE) [153]. Most of these genes involved in PPAR*γ* signaling, Adipoq, Scd1, Nr1h3, Fabp5, Scd2, and Pparg decreased upon GSE treatment, whereas the expression of Ppargc1a was elevated [153, 154]. However, lipid metabolism-associated genes Mlxp1, Stat5a, Hsl, Plin1, and Vdr were downregulated. Thus, GSE containing resveratrol has been claimed to modulate key transcription factors including peroxisome proliferator-activated receptor, CCAAT/enhancer-binding proteins, and their target genes (FAS, aP2, SCD-1, and LPL). It remains to be determined whether a novel regulator of mammalian target of rapamycin complex 1 (mTORC1) plays an important role in the stilbene-mediated adipocyte differentiation of 3T3-L1 preadipocytes and potential prevention of obesity as found for other polyphenols [155].

Lipolysis regulates the key metabolic roles in the formation of adipose tissue size, weight, and obesity and two enzymes, adipose triglyceride lipase (ATGL) and hormone-sensitive lipase (HSL), are involved in the lipolytic activity. HSL is active against diglycerines while ATGL selectively act on the first step in the triglycerine hydrolysis resulting in the formation of diglycerines and free fatty acids [156]. Stilbene compounds can modulate lipogenesis in many ways; for example, using knockout mice it was found that resveratrol regulates lipolytic activity in human and murine adipocytes, as well as in white adipose tissue from mice mainly via ATGL at transcriptional and posttranscriptional levels [156]. SIRT1 and FOXO1** (**Forkhead box protein O1) involve the regulation of lipolysis so that SIRT1 probably affects the acetylation status and functional activity of FoxO1 so that it may directly bind to the ATGL promoter [157]; thus it apparently regulates ATGL gene transcription. Two other studies [158, 159] provide support for the important role of SIRTI and FOXO1 in the regulation of transcriptional expression of ATGL in adipocytes. Gene expression patterns of two human tissue samples (subcutaneous abdominal adipose tissue SAT and visceral adipose tissue VAT) derived from nonobese and class III obese subjects were recently analyzed [160]. Interestingly, adiponectin expression was lower only in VAT of obese subjects while FOXO1 and PPAR*γ* levels were decreased in VAT of both groups. However, there was no difference with regard to the SIRT1 levels in VAT or SAT in both groups.

AMPK is an important regulator of energy metabolism and thus it is a key component in obesity regulation. There is an abundance of data indicating that resveratrol can activate AMPK for example [145]. Resveratrol may activate AMPK via inhibition of ATP production but this action seems to be dependent on high doses of resveratrol [161]. Importantly, it was shown that resveratrol increased cAMP levels by competitively inhibiting a number of cAMP phosphodiesterases (PDEs) [105, 161], which degrade cAMP; this suggests that PDE4 inhibitors may be used to develop drugs or special food supplements for therapeutic options for obesity management.

Obesity is known to be related with chronic low-grade inflammation condition leading to the production of a number of inflammatory cytokines, chemokines, and prostaglandins which eventually can lead to the development of insulin resistance. Consequently, targeting specific stilbene compounds to prevent or inhibit the inflammation cascade may be attractive means to reduce obesity and T2D. There is an extensive literature that different stilbenes including pinosylvin, piceatannol, and resveratrol can reduce the development of inflammatory cytokines [9, 162, 163]. Stilbenes and GSEs appear to mediate the attenuation of inflammation and insulin resistance apparently by suppressing the activation of extracellular signal-regulated kinase (ERK), c-Jun N-terminal kinase (JNK), and NF-*κ*B (nuclear factor kappa-light-chain-enhancer of activated B cells) [164, 165], but the anti-inflammatory property may also involve the SIRT1 pathway [166]. In rats, resveratrol may mediate body-fat reduction also via the modulation of thermogenesis as UCP protein was increasingly expressed after resveratrol treatment in the important thermogenic levels [167].

Collectively,* in vitro* and animal studies suggest that stilbenes mediate their antiobesity action via several mechanisms including the inhibition of lipid synthesis in adipocytes, modulation of lipolysis, modulation of apoptosis or mTORC1, and activation of AMPK via inhibition of ATP production as well as reducing inflammation and oxidative stress in the target tissue. The development of specific weight management food products focusing at multiple molecular targets may be a promising avenue for enhancing the antiobesity effect, but this approach may benefit from the combination of distinct polyphenols in the product [168, 169].

### 5.2. Type 2 Diabetes

Type 2 diabetes mellitus (T2D) is a rapidly and globally increasing complex metabolic disorder associated with elevated insulin resistance, decreased insulin secretion, impaired insulin signaling, hepatic *β*-cell dysfunction, abnormal glucose and lipid metabolisms, elevated inflammatory burden, and increased oxidative stress. Drugs are widely used to maintain the normal blood glucose level to prevent the development of hyperglycemia which may lead to a number of diabetic complications. It is well documented that diet is one of major risk factors for the development of metabolic disorders leading to T2D, and increasing data suggests that a diet rich in polyphenols and fiber may lower the incidence of T2D by reducing the major predisposing metabolic risk factors.

A considerable amount of* in vitro* and preclinical data implicates that stilbene compounds may lower risk factors behind T2D via several mechanisms. There are recent animal trials suggesting that stilbene compounds, particularly resveratrol, may reduce blood glucose levels in mice, rats, and rodents with hyperglycemia and also modulate insulin levels. In a recent mice trial both low (0.005%) and high levels (0.02%) of resveratrol diet given for six weeks significantly decreased blood glucose, plasma free fatty acid, triglyceride, and apo B/apo AI levels and increased plasma adiponectin levels [170]. Decreased glucose levels were found to be associated with activated levels of AMPK and its downstream targets leading to decreased blood HbA1c levels, hepatic gluconeogenic enzyme activity, and hepatic glycogen. However, only after high dose resveratrol supplementation, there were increases in levels of insulin, pancreatic insulin protein, and skeletal muscle GLUT4 protein. Furthermore, there is a report that, although low dose (30 mg/kg daily for two weeks) treatment could lower fasting glucose level, the resveratrol treatment enhanced insulin action only under insulin-resistant conditions and the treatment efficacy was found to depend on the target tissue and its metabolic stage [171]. In an experiment in T2D model db/db mice another stilbene treatment (piceatannol) was noted to enhance glucose uptake, AMPK phosphorylation, and GLUT4 translocation to the plasma membrane in conditions of insulin absence [172]. Interestingly, they found that piceatannol suppressed the elevations in blood glucose levels in the early stages and improved the impaired glucose tolerance in the later stages in db/db mice. In a rat trial, it was shown that resveratrol treatment inhibited HFD-induced glucose intolerance and insulin resistance in ovariectomized rats [173]. Furthermore, increased insulin-stimulated glucose uptake was demonstrated in isolated soleus muscle* in vivo* and in C2C12 myotubes* in vitro* with mechanism attributed to enhancement of GLUT4 translocation to the plasma membrane rather than increasing GLUT4 protein expression. Interestingly, they were able to show that CAV-3 protein (caveolin family proteins) expression was increased after resveratrol treatment, which contributed to GLUT4 translocation.

*α*-Glucosidase and *α*-amylase are digestive enzymes participating in starch and disaccharide degradation. By inhibiting the action of these enzymes with drugs (e.g., acarbose and voglibose) it is possible to slow down glucose absorption from intestine to bloodstream and hence to reduce postprandial hyperglycemia. In addition, many polyphenols are capable of inhibiting *α*-glucosidase and *α*-amylase enzyme activity. Numerous vegetable, herbal, fruit, and berry extracts especially those rich in flavonols, ellagitannins, anthocyanins, phenolic acids, and their derivatives have demonstrated* in vitro* inhibitory activity with respect to both *α*-glucosidase and *α*-amylase [174–176]. However, little is known about the impact of stilbenes on these molecular targets. Some stilbenoids and stilbene glycosides (e.g., 4′-*O*-methyl piceid, rhapontin, rhapontigenin, and desoxyrhapontigenin) from rhubarb (*Rheum palmatum* and* Rheum emodi Wall. ex Meissn.*) have been observed to inhibit and modulate *α*-glucosidase activity [177, 178]. In addition,* trans*-stilbenes resveratrol and rumexoid from the roots of buckwheat* Rumex bucephalophorous* and monomeric and dimeric stilbenoids (e.g., piceatannol, resveratrol, and scirpusin) from the seeds of palm* Syagrus romanzoffiana* have revealed inhibitory activity against *α*-glucosidase [179, 180]. For example, piceatannol dimers,* trans* double bond, tetrahydrofuran ring, and free adjacent phenolic dihydroxyls may be important features in the inhibitory properties [181]. Based on* in vitro* assay and docking studies, resveratrol-3-*O*-glucosidase from grape skin extracts has been speculated to bind to *α*-amylase in an inhibitory manner [182]. It has also been postulated that biotransformation, for example, dimerization, of stilbene compounds may be a way to enhance their efficacies as antihyperglycemic agents [181].

Insulin suppresses lipolysis in both transcriptional and posttranscriptional levels in adipose tissue. Apparently, insulin signaling acutely inhibits beta-adrenergic signaling by decreasing intracellular cyclic AMP (cAMP) levels and the rate of lipolysis [183]. Moreover, in the case of insulin resistance and T2D, attenuation of lipolysis by insulin action is impaired leading to an increased rate of lipolysis and enhanced release of free fatty acids (FFA) in the circulation [183]. A very interesting novel protective mechanism of resveratrol against aging-related metabolic degeneration was described by Park et al. [105]. They hypothesized that the metabolic impact of resveratrol results from competitive inhibition of cAMP-degrading phosphodiesterases. Apparently elevated cAMP levels can activate a cAMP effector protein (Epac1), leading to higher concentrations of intracellular Ca^2^; eventually this will lead to the increasing uptake of resveratrol and elevated NAD^+^ levels and increased activity of SIRT1. It has therefore been postulated that the inhibition of PDE4 activity via bioactive compounds may protect from or ameliorate the symptoms of metabolic diseases associated with aging such as T2D.

There is considerable data highlighting the vital role of oxidative stress as an important risk factor in development of T2D. Activation of antioxidant defence and phase II enzymes is a key mechanism to protect cells from the oxidative damage involved in age-related diseases such as T2D. By using methylglyoxal (MG) as a tool to induce insulin resistance in HepG2 cells, Cheng et al. [80] demonstrated that resveratrol activated ERK pathway but not the p38 or JNK pathways, and this eventually led to Nrf2 nuclear translocation and elevation of HO-1 and glyoxalase expression levels. Furthermore, they found that resveratrol significantly elevated glucose uptake and protected HepG2 cells against MG-induced insulin resistance. Recently, when a 20 mg/kg daily dose of resveratrol was administrated for 12 weeks to db/db mice, improved glucose tolerance, attenuated *β*-cell loss, and reduced oxidative stress were documented [184]. The protective function of resveratrol against cellular oxidative stress through the SIRT1-FOXO pathway under high-glucose (HG) conditions was recently demonstrated [185]. Under HG conditions* in vitro*, SIRT1 and FOXO3a were significantly decreased compared with normal glucose conditions and this was reversed by resveratrol treatment concomitant with the reduction in HG-induced superoxide production and p47phox. Thus, the data suggests that resveratrol decreases HG-induced superoxide production via upregulation of SIRT1, induction of FOXO3a, and inhibition of p47phox in monocytes. Although a vast number of* in vitro* and animal studies hint at the vital role of oxidative stress in T2D, more clinical data, however, are needed to confirm this hypothesis.

T2D is also an inflammation-related disease: expanded visceral adipose tissue may disturb insulin signaling pathways by excreting inflammatory factors. It has long been known that anti-inflammatory agents may be one therapeutic means to reduce the risk of developing this disease. A wide body of data indicates that stilbene compounds demonstrate anti-inflammatory properties* in vitro*.

Overall, the accumulated data suggest that stilbene-like polyphenols can modulate blood glucose and insulin levels and reduce oxidative stress and inflammation meaning that this represents a rational molecular target for novel target-specific food product development.

*Beneficial Role of Stilbenes on Diabetic Vascular Diseases.* Diabetes has been shown to associate with the development of cardiovascular diseases (CVD) such as atherosclerosis [186]. In CVD, vascular inflammation, increased platelet aggregation, and decreased levels of vascular nitrogen oxide (NO) production disturb the functions of the vascular endothelium [187]. In vascular endothelium, oxidative stress decreases NO bioavailability and in the presence of superoxide anion (O^2−^) it increases the formation of peroxynitrite (ONO_2_^−^), a powerful oxidant [106]. Although NO is a free radical, it is also an important cellular signaling molecule and a major regulator of vascular functions such as vascular tone, platelet aggregation, and vascular proliferation [106, 187]. In the vascular endothelium, NO is synthesized by endothelial nitric oxide synthase enzyme (eNOS) with the assistance of tetrahydrobiopterin (BH4), an essential cofactor of eNOS [116]. Importantly, it has been shown that in CVD and diabetes, decreased NO levels are a result of ROS induced elimination of BH4 stores in vascular endothelium [106, 108, 188].

The cardioprotective functions of stilbene compounds such as piceatannol and resveratrol have been intensively studied in animals [145, 189] and in humans [112, 190–192]. Several molecular targets for stilbenes with cardioprotective activity such as cyclooxygenases (COX-1 and COX-2), eNOS, Nrf2, ERs, and SIRT1 have been proposed [188]. Resveratrol and piceatannol can support endothelial functions such as vasorelaxation by increasing NO production and by reducing ROS via eNOS and NADPH enzymes, respectively [193–196]. The beneficial effects of stilbenes in CVD are also mediated via regulation of cellular BH4 homeostasis. It has been shown that resveratrol decreases BH4 degradation in parallel with the induction of BH4 synthesis via GTP cyclohydrolase 1 (GCH1) [116]. Apparently, the anti-inflammatory functions of stilbenes can also be mediated via the COX enzymes [197] as well as the Toll-like receptor 4 (TLR4) [198].

### 5.3. Neurodegenerative Diseases of the Aging Eye and Brain

AMD and AD share common features with the neurodegenerative aging diseases, that is, abnormal accumulation of insoluble protein aggregates (lipofuscin, drusen, and AD plaques), perturbation of autophagy clearance system, and increased cellular status of oxidative stress and inflammation [199–203]. Moreover, increased levels of labile cellular iron, a powerful generator of ROS involved in oxidative stress, have been observed in both diseases [204, 205]. It is noteworthy that, although these diseases have similarities, the genetic component of AMD and AD seems to be specific for the disease. In this section, the characteristics of AMD and AD, the beneficial functions of stilbenes, and associated cellular mechanisms are discussed.

#### 5.3.1. Age-Related Macular Degeneration (AMD)

Age-related macular degeneration (AMD) is the leading cause of blindness in an aging population affecting the life of 30–50 million individuals [206]. AMD is a multifactorial, progressive degeneration of the central retina with two distinct subforms [207]. The atrophic form (dry AMD) with a prevalence of 85–90% represents a major healthcare burden since no effective cure is available. The wet form of AMD (prevalence 10–15%) with choroidal neovascularization and leaky blood vessels under the macula is more severe and has faster progression. AMD initiates from the RPE, eventually leading to degeneration of photoreceptors.

In AMD patients, retinal changes such as the formation of extracellular deposits (drusens) [208], accumulation of RPE lipofuscin [209], chronic inflammation [210], impaired autophagy [211], and neovascularization [212] are frequently observed. In addition to aging, genetic component, smoking, extensive light exposure, and decreased RPE pigmentation are known to be risk factors for AMD [213, 214]. In particular, the probability of increased chronic oxidative stress triggered by several factors unique for the eye seems to play central role in development of AMD [215]. First, RPE is located in exceptionally oxygen rich environment next to the choroidal vasculature network [215]. Second, due to continuous phagocytosis of photoreceptor outer segments (POS), RPE cells are repeatedly exposed to lipid peroxidation products [216] and to the phototoxic lipofuscin intermediate, bisretinoid A2E [217, 218]. Third, during its lifespan, RPE is exposed to intense stress and photobleaching of RPE melanin caused by sunlight and UV-radiation [214, 219]. In healthy RPE tissue, melanin is a potent scavenger of free radicals, which also inhibits lipid peroxidation, absorbs UV-radiation, and chelates metals such as labile iron [220, 221]. Labile iron is capable of inducing a Fenton reaction in cells, which is a powerful generator of free radicals and oxidative stress [205, 222, 223]. Reduced levels of RPE melanin pigment [219, 224–226] are commonly observed in AMD patients with concurrent increase of cytotoxic levels of labile iron in the RPE [204]. In addition to the devastating general actions of ROS in cells, increased levels of oxidative stress may specifically disturb fundamental functions of the RPE such as POS phagocytosis [227], visual cycle [93], and the integrity of the RPE barrier functions [228].

The reduced antioxidant capacity in the RPE is known to associate with age. For instance, data from mouse models indicates that the Nrf2 system declines with age subjecting RPE cells to oxidative stress [229]. It seems that Nrf2 is involved in the maintenance of retinal functions in general as revealed by current data obtained from Nrf2 knockout mouse model showing that perturbation of the Nrf2/ARE pathway has a remarkable role in development of age-related signs in retina AMD [71, 230]. Nrf2 knockout mouse seems to display all of the typical hallmark retinal changes encountered in AMD such as drusens, lipofuscin, choroidal neovascularization (CNV), and changes in RPE pigmentation. Experiments with Nrf2 deficient mice indicate that Nrf2 is also involved in reducing the chronic inflammation in the eye [231]. After inflammation, induced by lipopolysaccharide (LPS), Nrf2 deficient mice displayed increased levels of inflammation markers (ICAM, IL-6, TNFa, MCP-1, COX-2, and iNOS) in the retina in comparison to their wild-type counterparts.

It is claimed that polyphenolic compounds can exert a protective effect against the stress associated with aging of retinal cells. In particular, the induction of phase II enzymes via the Nrf2 pathway seems to play a key role in this defence system. For instance, pinosylvin was revealed to protect ARPE-19 cells (human RPE cell line) against oxidative stress mediated via the Nrf2 pathway by inducing HO-1 expression [232] and quercetin reduced the levels of inflammation markers IL-6 and IL-1*β* after oxidative stress induction in ARPE-19 cell line [233]. Similarly, hydroxytyrosol, a phenolic compound present in olive oil and red wine, has been demonstrated to activate Nrf2, HO-1, NQO-1, GCL, GSH, and p62 expression in ARPE-19 cells, and interestingly, GSH production was partially mediated via induced p62 expression [234]. Convincing evidence indicates that accumulation of p62 due to an impairment of the autophagy process is associated with degeneration of RPE cells [90]. Impaired autophagy clearance has been shown to associate with AMD [90, 212]. There is growing data that polyphenols can also modulate autophagy clearance mediated via the cAMP and AMPK pathways (see Section 4.3) [100, 235].

There are interesting results indicating that polyphenols can also influence the secretion of specific growth factors associated with AMD and other retinal diseases such as diabetic retinopathy. In a long-term trial of a small group of elderly AMD patients, daily administration of a polyphenol supplement containing 100 mg resveratrol, quercetin, apigenin, ferulic acid, and so forth exerted a beneficial effect on retinal integrity and anti-VEGF activity as well as an improvement of visual function [236]. In mice, resveratrol has been shown to suppress angiogenesis [237]. Similarly, resveratrol was able to decrease oxysterol induced VEGF secretion in ARPE-19 cells [238]. It is noteworthy that cigarette smoke is a major risk factor in AMD [230]. Cigarette smoke contains abundance free radicals such as hydroquinone (HQ) and these can decrease the levels of antiangiogenic PEDF accompanied by a simultaneous increase in the VEGF levels in RPE cells of smoking AMD patients [239]. This is feasible based on evidence detailing the role of PEDF as an inhibitor of choroidal neovascularization (CNV) in the retina [117, 240]. A recent study conducted in ARPE-19 cells revealed that 10 *μ*M resveratrol was able to prevent platelet-derived growth factor (PDGF) induced RPE cell proliferation and migration which are common phenomena in AMD, diabetic retinopathy, and proliferative vitreoretinopathy [241]. The beneficial effects of resveratrol have also been demonstrated in animal models where the animals were subjected to different retinal injuries such as retinal detachment [240], retinal ischemic injury [242, 243], light-induced retinal degeneration [244], endoplasmic reticulum stress related vascular degeneration [245], and retinal ganglion cell degeneration associated optic nerve injury [246]. Moreover, the beneficial effects of resveratrol against inflammation have been observed in mouse models. For instance, the activation of SIRT1 and the decreased nuclear localization of NF-*κ*B achieved by resveratrol were associated with reduced oxidative stress and a decrease in inflammation in the mouse retina [247]. A similar type of action was found with the resveratrol analog, piceatannol in the retina. In rodents, this compound has been shown to suppress endotoxin induced ocular inflammation [248] as well as Toll-like receptor 4 (TLR4) mediated inflammatory response and retinal damage occurring after retinal ischemia [249].

Taken together, polyphenols seem to exert numerous beneficial effects in retinal cells and thus they display a potential for the prevention of retinal diseases such as AMD. In addition to their direct antioxidant activity, polyphenols seem to display beneficial effects in the eye through anti-inflammatory activity and activation of autophagy as well as by induction of phase II enzymes via the Nrf2 pathway. Thus one can speculate that polyphenols may support integrity of the retina by controlling the expression and secretion of many of the growth factors such as VEGF, PEDF, and PDGF involved in neovascularization and cell proliferation.

#### 5.3.2. Alzheimer's Disease (AD)

Alzheimer's disease (AD) is a devastating neurodegenerative disorder exhibiting synaptic changes and neuronal loss in the hippocampus and cerebral cortex in the central areas of the brain involved in memory and cognition. Accumulation of extracellular plaques of amyloid *β* (A*β*) peptide and aggregation of microtubule-associated protein tau into insoluble intracellular neurofibrillary tangles are the characteristic hallmarks of AD. About 2.5% of the US population carries two genetic risk genes for ApoE4, the cholesterol-carrying protein, which increases the risk of developing AD by about 10-fold [250]. Despite intensive research and drug development, there is still no effective therapy against AD, and at present preventive approaches are thought to be the best way to address this growing public health problem. There are epidemiological studies indicating that the consumption of phenolic-containing berries, fruit, and vegetables can lower the risk of AD [3, 251]. For example, individuals drinking three or more glasses of fruit or vegetable juice per week have been shown to lower by over 50% their risk of AD in comparison to individuals who consumed less than one serving per week [251]. The protective activity conferred by the bioactive polyphenols in the juice is likely to be a result of multiple-target properties such as impaired insulin and insulin-like growth factor (IGF) signaling, A*β* and tau-protein accumulation, synaptic disconnection, and impaired energy in addition to limiting damage due to of oxidative stress and inflammation.

Since polyphenols have difficulties passing through the blood-brain barrier following oral administration, the resulting low concentration of polyphenols in the brain tissue has been thought as limiting their use against Alzheimer's disease, but it was recently shown that resveratrol, and particularly resveratrol metabolites, can reach such concentrations in the brain capable of achieving beneficial physiological changes [7, 47, 252]. For example, a higher concentration of resveratrol was achieved in the rat brain tissue when the compound was dispensed in lipid-core nanocapsules [47]. Importantly, the enhanced penetration of resveratrol into the brain was found to protect tissue from the deleterious effect of A*β*1-42 and the subsequent impairment of memory functions more effectively than resveratrol treatment without lipid-core nanocapsulation. Furthermore, the polyphenol metabolite, quercetin-3-*O*-glucuronide, was found to significantly reduce the generation of A*β* peptides by primary neuron cultures obtained from the Tg2576 AD mouse model [252]. Interestingly, quercetin-3-*O*-glucuronide was also capable of interfering with the initial protein-protein interaction of A*β* (1–40) and A*β* (1–42) necessary for the formation of the neurotoxic oligomeric A*β* species [252]. These are important findings since it is known that A*β*, which is released after sequential cleavage of amyloid precursor protein (APP) by *β*- and *γ*-secretases, is a key participant in AD pathogenesis [253]. Tau protein is known to be abnormally hyperphosphorylated in AD and aberrant tau phosphorylation contributes to the neuropathology of AD. Administration of polyphenol-rich GSE has been shown to interfere with the assembly of tau peptides into neurotoxic aggregates suggesting that polyphenols such as stilbenes can directly modulate the aggregation process of tau [254]. Interestingly, feeding mice for four months with a protein restriction (nonessential amino acid) based diet achieved a cognitive improvement and reduced pathological changes, associated with altered tau phosphorylation and disturbed levels of IGF-1 [255]. This observation suggests that provision of polyphenols and a protein restriction diet may mediate their neuroprotective action via common molecular mechanisms. There are recent findings indicating that only the apolipoprotein ApoE4 allele significantly decreases the ratio of soluble amyloid precursor protein alpha (sAPP*α*) to A*β* and is able to reduce SIRT1 expression resulting in markedly differing ratios of the levels of neuroprotective SirT1 to the neurotoxic SIRT2 as well as also triggering Tau and APP phosphorylation [250]. Stimulation of innate immunity via the Toll-like receptors such as TLR9 has been reported to effectively reduce the amyloid burden [256], but it remains to be determined whether stilbene compounds have sufficient efficacy to activate innate immunity.

Brain-derived neurotrophic factor (BDNF) plays a key role in brain cell development, growth, and survival since this growth factor promotes synaptic plasticity in the hippocampus. For example, BDNF mediates neuroprotective and cognitive function via inhibiting food intake and increasing energy expenditure in the hypothalamus [257]. There is some data indicating that stilbenes may directly modulate brain synaptic plasticity. Treatment of rats for 3, 10, and 30 days with resveratrol significantly and dose-dependently elevated the levels of BDNF mRNA expression in hippocampal tissue suggesting that the resveratrol mediated neuroprotective impact may be related with activation of the BDNF pathway [258]. Recently, grape powder extract was found to prevent oxidative stress-induced anxiety, memory impairment, and hypertension in rats by regulating also brain CREB and BDNF levels [259]. Furthermore, blueberry-fed animals exhibited a faster rate of learning and better spatial memory performance as compared to those on the control diet providing further evidence that polyphenols may mediate their neuroprotective action via BDNF [260]. Importantly, it was also observed that the improved behavioral performance was associated with increases in total CREB and elevated levels of pro- and mature BDNF in the hippocampus. Recent important findings have indicated that the monomeric proanthocyanidin metabolites seem to be the key PAC metabolite that is able to attain concentrations of ~400 nM in brain and improve cognitive function [7]. They further revealed that one of the epicatechin metabolites, 3′-*O*-methyl-epicatechin-5-*O*-*β*-glucuronide, could enhance synaptic plasticity through mechanisms associated with CREB signaling.

Autophagy, the lysosomal mediated degradative pathway for proteins and organelles, may be essential for the survival of mature neurons, but the underlying mechanisms remain to be elucidated. However, Marambaud and coworkers reported that resveratrol could not inhibit A*β* production, since it had no apparent effect on the A*β*-producing enzymes beta- and gamma-secretases, but instead it promoted intracellular degradation of A*β* via a mechanism involving the proteasome [261]. Subsequently considerable data have accumulated that autophagy is involved in the resveratrol mediated protection against oxidative stress, inflammation, and associated cardiovascular, cancer, and neurological diseases [262]. Resveratrol-mediated autophagy mechanisms may be dose-dependent [263], but their importance in neuroprotection, however, still remains to be clarified.

There is a growing body of experimental data strongly supporting the hypothesis that CR is a significant way to extend longevity and delay many age-related diseases review by [143, 264, 265]. The protective action of CR is potentially mediated via a reduction of inflammation and oxidative stress, but recent data have highlighted the possibility that CR may modulate also critical signaling pathways such as IGF-1/insulin signaling, sirtuin, AMPK, and mTOR pathways [255]. These metabolic pathways are considered as key risk factors affecting brain health and AD. However, one recent report suggested that part of the beneficial metabolic impacts may not simply be due to general CR, but also the food ingredients in the CR diet may play a role since those individuals consuming a CR diet had high intakes of vegetables, berries, and fruits which contain substantial amounts of bioactive compounds [265]. Stilbenes such as resveratrol are considered to exhibit to some degree of CR mimetic properties via the action on sirtuin. By using SAMP8 (Senescence-accelerated mouse mice), it was revealed that long-term resveratrol treatment could reduce the cognitive impairment by reducing the amyloid burden and tau hyperphosphorylation [266]. It was further demonstrated that resveratrol activated AMPK pathways and prosurvival routes such as SIRT1* in vivo*. However, in the same SAMP8 mice model, resveratrol and another stilbene, pterostilbene, did not increase SIRT1 expression although markers of cellular stress and inflammation and reduced AD pathology were positively modulated by pterostilbene but not resveratrol [8]; hence, pterostilbene's higher bioavailability (i.e., better than resveratrol) may have important protective implications.

There is emerging evidence suggesting that AD is fundamentally a metabolic disease and brain glucose utilization and responsiveness to IGF stimulation may play key roles behind neuronal loss, loss of synaptic connections, tau hyperphosphorylation, and A*β* accumulation [267]. Thus, suppression of energy expenditure by modulation AMPK, glucose transport, and the insulin pathway via stilbene-like polyphenols may represent a promising avenue to delay the onset of AD and slow disease development. Importantly, resveratrol was able to activate AMPK in neuronal cells* in vitro* as well as in the brain and it enhanced activation of mitochondrial biogenesis in an AMPK-dependent manner [268]. There are several animal experiments indicating that supplementation with stilbenes (resveratrol and piceatannol) can enhance glucose uptake, AMPK phosphorylation, and GLUT4 translocation (see diabetes section), but very few human trials have been performed. Interestingly, when mice (C57BL/6 Jl) were fed with HFD (high-fat diet) supplemented with resveratrol for 20 weeks, there were signs of reduced insulin resistance, lower levels of tumor necrosis factor-*α*, and Iba-1 in hippocampus as well as improvements in the normal memory deficits in HFD-fed mice [269].

Taken together, there is an impressive body of* in vitro* and animal data to suggest that stilbenes mediate their neuroprotective action via several mechanisms, that is, through the modulation of generation of A*β* and tau peptides, modulation of brain synaptic plasticity via BDNF, modulation of brain energy expenditure via AMPK, glucose transport, and insulin pathway, and reducing inflammation and oxidative stress burden. Experiments in mice models of ADs have indicated that polyphenol based diets can alleviate the spatial working memory deficit and some other cognitive traits. These studies provide new ideas for the development of novel target-specific medicinal foods and dietary supplements for the ever increasing elderly population.

## 6. Lessons from Preclinical and Clinical Trials

There is convincing epidemiological data emphasizing that a diet rich in vegetables and fruits confers many health benefits. There is also increasing preclinical data obtained in various animal models indicating that polyphenols such as stilbenes can target critical sites involved in the disease process and ultimately alleviate disease outcomes. However, very few well-planned clinical trials are available which would have provided solid evidence for the health benefits of stilbenes for humans.

Recently, a very interesting long-term trial determining the impact of resveratrol supplementation on adipose tissue, insulin signaling, and inflammatory response was reported using rhesus monkeys as the model [270]. Two-year supplementation with 80 and 480 mg/day for the first and second year, respectively, was found to decrease adipocyte size, increase SIRT1 expression, decrease NF-*κ*B activation, and improve insulin sensitivity in visceral, but not subcutaneous WAT (white adipose tissue) in the HFS-fed animals. Furthermore, the impact of resveratrol supplementation on obesity has been evaluated in a few clinical trials. For example, 11 healthy obese men were supplemented with 150 mg/day resveratrol in a randomized double-blind crossover study for 30 days [271]. Resveratrol treatment activated AMPK, increased SIRT1 and PGC-1*α* protein levels, improved muscle mitochondrial respiration on a fatty acid-derived substrate, elevated intramyocellular lipid levels and decreased intrahepatic lipid content, reduced levels of circulating glucose, triglycerides, alanine-aminotransferase, and decreased the levels of inflammation markers. Furthermore, declines were observed in the systolic blood pressure, adipose tissue lipolysis, and plasma fatty acid in the postprandial stage. Interestingly, resveratrol supplementation for 30 days in obese men modulated postprandial glucagon responses [105]; it is known that glucagon and related hormones may stimulate adenylate cyclases (AC), resulting in increased cAMP production [161].

In a long-term human trial, molecular changes in peripheral blood mononuclear cells (PBMCs) associated with the one-year daily intake of a resveratrol grape extract (GE) and GE enriched 8 mg of resveratrol (GE-RES) in hypertensive male patients was studied [272]. This data revealed that GE or GE-RES did not affect body weight, blood pressure, glucose, HbA1c, or lipids, but a significant reduction in the levels of ALP (alkaline phosphatase) and IL-6 was recorded. The expression of the proinflammatory cytokines CCL3, IL-1*β*, and TNF-*α* was significantly reduced and that of the transcriptional repressor LRRFIP-1 increased in PBMCs from patients consuming the GE-RES extract. Furthermore, a group of miRs (microRNA expression) involved in the regulation of the inflammatory response, that is, miR-21, miR-181b, miR-663, miR-30c2, miR-155, and miR-34a, were found to be highly correlated and altered in the group consuming the GE-RES for 12 months. Thus this human trial provided the first indication that long-term supplementation with a GE-RES could downregulate the expression of key proinflammatory cytokines suggesting that this polyphenol treatment may have a true beneficial immunomodulatory impact in humans suffering from hypertension [272]. A recent meta-analysis based on eleven human clinical trials (involving a total of 388 subjects) indicated that resveratrol supplementation could significantly reduce fasting glucose, insulin, hemoglobin A_1c_, and insulin resistance levels in participants with diabetes, but no significant impact on glycemic parameters was recorded in nondiabetic participants [4].

Epidemiological studies have indicated that greater intakes of fruit and vegetable juices [251] as well as higher intakes of blueberries or its component anthocyanidins [3] were associated with slower rates of cognitive decline. Consumption of the Mediterranean-type diet, which consists of foods such as vegetables, nuts, and fish and combines micro- and macronutrients with substantial amounts of bioactive polyphenols, has been also associated with decreased cognitive decline [273]. New prospective studies performed with the Mediterranean diet also provide evidence that not only can this diet slow down the progression of AD but it also reduces the risk of CVDs and other metabolic disorders [274]. However, the therapeutic value of stilbene compounds in the prevention of progression of AD remains to be demonstrated in long-term clinical trials with different stilbenes and variable doses. There are animal trials suggesting that different stilbene compounds together may provide enhanced protection above that obtained with the single compounds [275] perhaps through affecting different molecular targets. Thus, long-term human trials with different combinations of stilbenes may be a promising avenue for revealing the potential therapeutic value of stilbenes in AD management. Souvenaid, a currently marketed medicinal food product, has achieved some improvements in cognitive function in patients with AD in small human trials [276].

## 7. Conclusions

**Figure 3:**
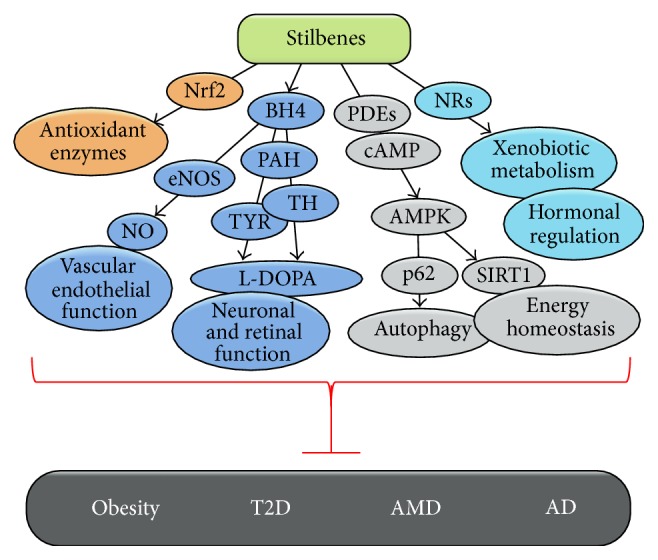
Schematic diagram showing the potential beneficial actions of stilbenes in prevention of age-related diseases.

There is emerging* in vitro* and preclinical data to indicate that stilbene compounds are capable of suppressing oxidative stress, inflammation, and energy expenditure as well as modulating the secretion of neurotropic factors. However, most of the available experimental data has concentrated on resveratrol and only limited research has been carried out with the other stilbene compounds. These other stilbenes such as pinosylvin, piceatannol, and pterostilbene may have higher biological activity than resveratrol and deserve to receive more scientific attention. The limited bioavailability, the low target specificity, and the rapid metabolism of stilbenes represent obstacles to achieving high enough concentrations of these compounds in plasma and the target tissue in order that they can exert their beneficial actions. However, recent investigations into the biological activity of polyphenol metabolites have been promising. Unlike drugs, stilbene-like polyphenols affect several key metabolic pathways (Figure 3), and promising therapeutic approaches may require focusing on multiple targets. This may lead to the development of next-generation functional type foods and supplements for slowing down the increasingly common diseases such as obesity, T2D, AMD, and AD.

## Figures and Tables

**Table 1:** Major stilbenes and their structures.

Plant	Compounds [reference]
Cocoa (*Theobroma cacao* L.)	Resveratrol [21]
Grape (*Vitis vinifera* L.)	Piceatannol, resveratrol [22]
Hop (*Humulus lupulus* L.)	Resveratrol [23]
Peanut (*Arachis hypogaea* L.)	Resveratrol [24]
Pinaceae trees (pines)	
*Picea* Mill.	Isorhapontigenin, piceatannol [25]
*Pinus* L.	Pinosylvin [16]
Rhubarbs (*Rheum* L.)	Piceatannol, rhapontigenin, resveratrol [26]
Strawberry (*Fragaria x ananassa* Duch.)	Resveratrol [27]
Sugar cane (*Saccharum* spp.)	Piceatannol, resveratrol [28]
Tomato (*Lycopersicon esculentum* Mill.)	Resveratrol [29]
*Vaccinium* berries	
Bilberry (*V*. *myrtillus*)	Resveratrol [30]
Cranberry (*V*. *macrocarpon*)	Resveratrol [30]
Highbush blueberry (*V*. *corymbosum*)	Piceatannol, resveratrol [30], pterostilbene [31]
Wines	
Red	Piceatannol, resveratrol [32]
White	Resveratrol [33]

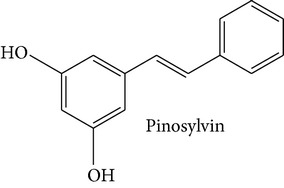	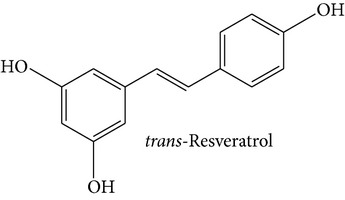
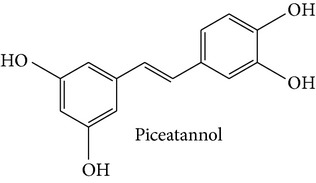	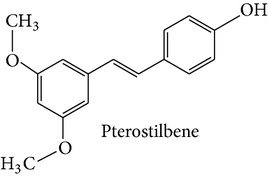
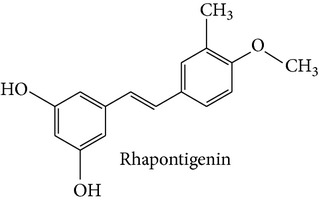	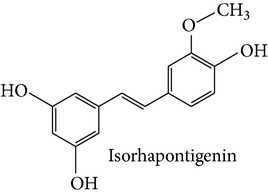

**Table 2:** Selected Nrf2 target gene candidates in human associated with defence against oxidative stress and age-related diseases.

Target gene	Function/role in defence against oxidative stress	Reference
Nrf2	Transcription factor, activator of detoxifying enzymes (autoregulation)	[73]
AhR	Regulator of xenobiotic metabolizing enzymes	[74]
HO-1	Cytoprotection, catabolize heme	[75]
GSTP1	Antioxidant enzyme, xenobiotic metabolizing enzyme	[76]
NQO1	Antioxidant enzyme, xenobiotic metabolizing enzyme	[77–79]
CBR3	Metabolizing enzyme of carbonyl compounds	[80]
UGT1A8, A10	Glucuronidation of xenobiotics	[81]
GCS	Glutathione biosynthesis	[82]
TRX	Antioxidant enzyme, protein redox regulation	[83]
SLC7A11	Transports cysteine, a precursor of antioxidant glutathione	[52, 84]
SLC48A1	Heme transporter	[85]
AMBP	Heme binding, free radical scavenger	[72]
		[85]
ABCB6	Mitochondrial porphyrin (heme) transporter	[85]
FECH	Heme biosynthesis, chelates ferrous iron	[72, 85]
TBXAS1	Thromboxane A2 synthesis (cytochrome P450 family)	[85]
IL-6	Inflammation, proinflammatory cytokine	[52, 86]
Bcl-2	Antiapoptotic protein	[87]
p62	Adaptor protein, proteasomal clearance, autophagy	[52, 66]

ABCB6: ATP-binding cassette subfamily B member 6, AhR: aryl hydrocarbon receptor, AMBP: *α*1-microglobulin/bikunin, Bcl-2: B-cell lymphoma 2 protein, CBR3: carbonyl reductase 3, FECH: ferrochelatase, GCS: *γ*-glutamylcysteine synthetase, GSTP1: glutathione S-transferase pi, HO-1: heme oxygenase-1, IL-6: interleukin-6, NQO1: NAD(P)H dehydrogenase, quinone 1, Nrf2: nuclear factor-erythroid-2-related factor-2, p62: sequestosome 1, SLC7A11: solute carrier family 7, member 11, SLC48A1: solute carrier family 48, member 1, TBXAS1: thromboxane A synthase 1, TRX: thioredoxin, and UGTs: UDP-glucuronosyltransferases.
